# Evaluation of a Simplified Upper Arm Device for Vacuum-Assisted Collection of Capillary Blood Specimens

**DOI:** 10.3390/diagnostics15151935

**Published:** 2025-07-31

**Authors:** Ulrich Y. Schaff, Bradley B. Collier, Gabriella Iacovetti, Mitchell Peevler, Jason Ragar, Nicolas Tokunaga, Whitney C. Brandon, Matthew R. Chappell, Russell P. Grant, Greg J. Sommer

**Affiliations:** 1 Diagnostic Devices Research and Development, Labcorp, Pleasanton, CA 94588, USA; 2 Center for Esoteric Testing, Labcorp, Burlington, NC 27215, USA

**Keywords:** microsampling, capillary blood collection, alternative phlebotomy method, pain-free phlebotomy, difficult venous access

## Abstract

**Background/Objectives:** Conventional blood collection can be challenging in a non-clinical or home-based setting. In response, vacuum-assisted lancing devices for capillary blood collection (typically from the upper arm) have gained popularity to broaden access to diagnostic testing. However, these devices are often costly relative to the reimbursement rate for common laboratory testing panels. This study describes the design and evaluation of Comfort Draw™, a simplified and economical vacuum-assisted capillary blood collection device. **Methods:** Comfort Draw™ was evaluated by 12 participants in a preliminary study and by 42 participants in a follow-up study. Metrics assessed included the following: vacuum pressure of the device, skin temperature generated by the Comfort Draw prep warmer, blood collection volume, and analytical accuracy (for 19 common serum-based analytes). **Results:** Acceptable blood volume (>400 µL) and serum volume (>100 µL) were collected by Comfort Draw in 85.5% and 95.1% of cases, respectively. Seventeen of the nineteen analytes examined were within CLIA acceptance limits compared to matched venous samples. Self-reported pain scores associated with Comfort Draw collection averaged 0.39 on a scale from 0 to 10. **Conclusions:** In this preliminary clinical study, Comfort Draw was found to be a valid and relatively painless method for collecting capillary blood specimens. The device’s simple design and lower cost could enable broader applications compared to more complex alternative capillary blood collection devices.

## 1. Introduction

Blood specimens for laboratory analysis are typically obtained via venous phlebotomy or by lancing capillary beds, usually via fingerstick or heel-stick. Venous phlebotomy is a demanding skill that requires a highly trained technician to locate and access suitable veins and is, therefore, only available in certain settings. Although conventional capillary blood collection techniques require less skill, recovering volumes of blood suitable for typical test panels (i.e., >100 µL) remains difficult and inconsistent [1]. Furthermore, blood collection from sensitive fingertips or difficult-to-locate veins can be a painful and anxiety-inducing experience for patients. Conventional blood collection methods are, therefore, challenging in non-clinical settings such as lightly staffed collection sites or the home. A reliable, easy-to-use, and relatively pain-free method of collecting blood specimens is needed to broaden the accessibility of diagnostic testing [2].

The traditional practice of wet cupping, or Al-Hijamah, in which a cup-shaped vessel is applied over an incision made on the body and blood is drawn out by inducing a vacuum, has been used for centuries throughout Southeast Asia, the Middle East, and Eastern Europe as a means of therapeutic bloodletting [3]. This ancient technique has recently been adapted in the form of various vacuum-assisted lancing devices for specimen collection such as Tasso+ (Tasso, Inc.; Seattle, WA, USA), TAP^®^ Micro Select (YourBio Health, Inc.; Medford, MA, USA), and RedDrop (RedDrop Dx, Inc.; Fort Collins, CO, USA). Such vacuum-assisted lancing devices work by creating one or more incisions on a part of the body with a low density of pain receptors (such as the lower back or upper arm), creating a partial vacuum over the wound, and directing the resulting capillary blood flow into an attached specimen collection tube [2]. These devices are typically used in conjunction with a warmer that is applied to the collection site for a few minutes to dilate capillary beds and further increase blood flow. The vacuum-assisted lancing devices have generally been found to be easy to use, nearly painless, and reliable in collecting 200–600 µL of capillary blood while producing valid results for multiple analytes of interest [4,5,6,7,8,9,10,11,12]. However, combining a vacuum-creating apparatus with a lancing mechanism results in a relatively complex mechanical device that can be costly relative to the reimbursement rate for common test panels. The relatively high cost of these devices limits their use to high-value testing and impedes more common applications. This study presents a new, simplified vacuum assist device, the Labcorp Comfort Draw™ (“Comfort Draw”), that enables use of economical conventional lancets to reliably obtain capillary blood specimens from the upper arm or other low-sensitivity locations on the body.

## 2. Materials and Methods

### 2.1. Device Design

**Figure 1:**
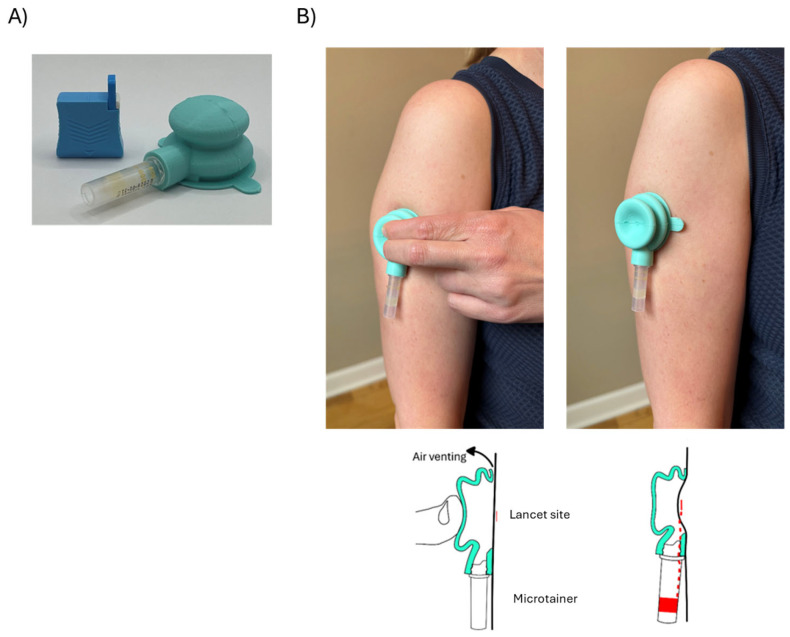
(**A**) Photo of Labcorp Comfort Draw™ and gentleheel^®^ lancet; (**B**) Photos (top) and cross-section schematic drawings (bottom) of Comfort Draw device on the upper arm showing operation of the device: positioned over the lancet site and under compression (causing venting of air from the bulb), and following compression (while in the vacuum phase); red dashed line indicates blood flow. Note: The device does not have an integrated adhesive layer. A detailed explanation of the collection process can be found in Supplemental Figure S1.

The Comfort Draw device was designed to enhance capillary blood collection when using conventional single-use lancets. The device is molded as a collapsible, half-bulb-shaped silicone rubber piece that interfaces with a removable BD Microtainer^®^ (Becton, Dickinson and Company; Franklin Lakes, NJ, USA) or other microvolume collection tubes. When compressed against the body on relatively flat skin surfaces such as the upper arm, air is expelled from the bulb, creating a partial vacuum when the bulb is released (Figure 1). The vacuum temporarily adheres the device to the skin by suction and greatly increases the capillary blood flow rate from a pre-applied lancet incision. Blood is directed into the BD Microtainer^®^ tube. For this evaluation, the gentleheel^®^ Newborn Heel Incision Device—Toddler (GRI Alleset; Flowery Branch, GA, USA) was selected as a lancet suitable for use with Comfort Draw. A structurally identical version of this lancet, intended for use on adults, has recently received FDA clearance [13].

### 2.2. Materials

Comfort Draw devices were molded from 2-part liquid silicone rubber with a hardness of 60 A Shore Durometer (Smooth-Sil™ 960; Smooth-On, Inc.; Macungie, PA, USA). As an accessory to Comfort Draw, custom body warmers were developed containing 50 mL of an aqueous sodium acetate solution sealed into flat vinyl pouches with a steel disk activator. The warmers were optimized to achieve and maintain a skin temperature approximating the optimum temperature identified in the literature (42 °C for more than 4 min) [14]. It was determined through sequential testing that a composition of 80–86% sodium acetate trihydrate in water was ideal for achieving the target temperature. The model tested in these studies used an aqueous solution of 81% sodium acetate trihydrate (Sigma-Aldrich; Saint Louis, MO, USA).

### 2.3. Vacuum Pressure and Skin Temperature Measurement

To assess the magnitude and reproducibility of vacuum generation, Comfort Draw was evaluated using an absolute pressure sensor (Digital Pressure and Altimeter Sensor Module MS580302BA01; TE Connectivity; Galway, Ireland) with a rated accuracy of ±0.25 kPa embedded in a modified Microtainer^®^. For each of the 6 subjects, the sensor was used to measure the internal pressure for a Comfort Draw device applied to the skin of the upper arm for the right and left sides of each subject. Devices were applied by an assisting technician. For each measurement, pressure was recorded immediately after activation and every 60 s thereafter for a total of 4 min. The difference between ambient pressure and internal pressure was used to calculate the relative vacuum pressure for each device.

The skin temperature generated by both the Comfort Draw prep warmer and a Medline Infant Heel Warmer (Medline Industries, LP; Northfield, IL, USA) when each was preconditioned at 22 °C and at 25 °C was evaluated by a thermocouple placed in contact with a subject’s upper arm skin, and directly under the warmer. Temperature was recorded 1, 2, 3, and 4 min after each warmer was activated, applied to the subject’s skin, and held in place manually by the subject.

### 2.4. Evaluation of Blood Collection Volume and Analyte Integrity

In a preliminary study, 12 subjects were recruited under IRB oversight (WCG IRB Protocol # 520100174; Approved 14 February 2024; WCG; Cary, NC, USA) to participate in a comparison between the Comfort Draw system and conventional fingerstick collection using the Unistik^®^ Touch lancet (Owen Mumford Ltd.; Woodstock, UK), both of which were used to collect blood into BD Microtainer^®^ SSTs (Becton, Dickinson and Company; Franklin Lakes, NJ, USA). Blood was collected, as described below, from each subject sequentially without a time gap between the two collections using each method in randomized order. Informed consent for participation was obtained from all subjects involved in the study.

Comfort Draw: A trained individual used the Comfort Draw system to collect the blood specimens. A target site on the upper arm of each subject was warmed using the prep warmer for 3 min by having the subject hold the prep warmer in place as directed, sterilization was performed with an alcohol pad, and then the gentleheel^®^ lancet (GRI Alleset; Flowery Branch, GA, USA) was applied and activated on the target site. A Comfort Draw device was centered on the incision and compressed to initiate vacuum. Capillary blood was collected into a BD Microtainer^®^ SSTs for up to 4 min following lancing or until the collected volume reached 600 µL (the nominal maximum fill line for the tube). (Simplified process: Figure 1; Detailed process: Supplemental Figure S1).

Fingerstick: A trained individual used the fingerstick lancet to collect a blood specimen. A target site on the fingertip of each subject was warmed using the prep warmer for 3 min, sterilization was performed with an alcohol pad, and then the lancet was applied and activated on the target site. Slight pressure was manually applied to the finger pad periodically (approximately 1 s on and 1 s off) to facilitate blood flow. The fingertip was held over the mouth of a BD SST Microtainer^®^, and blood was collected dropwise for up to 4 min following lancing or until the collected volume reached 600 µL.

Following collection, subjects were asked to self-rate perceived pain from the capillary collection process (for fingerstick and Comfort Draw) on a scale of 0 (no pain) to 10 (worst imaginable). The difference in perceived pain between fingerstick and Comfort Draw was statistically assessed via a paired Mann–Whitney test conducted using Prism 10 (GraphPad Software, Boston, MA, USA), with *p* < 0.05 being considered significant. The quantity of blood collected by each method was evaluated by comparing the weight of each filled Microtainer to the weight of a reference empty Microtainer and estimating blood density as 1.06 g/cc.

In a follow-up study, 42 subjects were recruited to participate in a clinical evaluation of the Comfort Draw system under the same IRB protocol. Informed consent for participation was obtained from all subjects involved in the study. The Comfort Draw system was operated by a trained individual to collect a capillary blood specimen from each subject, as previously described. Each subject had a reference blood specimen drawn immediately thereafter by standard venous phlebotomy into a BD Vacutainer^®^ SSTs (Becton, Dickinson and Company, Franklin Lakes, NJ, USA). Following collection, subjects were asked to self-rate perceived pain from the capillary collection process on a scale of 0 (no pain) to 10 (worst imaginable).

All Microtainers were photographed immediately after collection using a fixed-position camera to estimate blood volume and then allowed to clot for 30–60 min. Photographs were subsequently analyzed via ImageJ 1.54 (National Institutes of Health) by measuring the height in pixels of the blood layer in the tube relative to the optical position of the tube bottom, the 400 µL line, and the 600 µL line printed on the BD Microtainer^®^ SSTs. The blood volume was estimated from the image measurements by linear regression. Microtainers^®^ were then separated by centrifugation at 6000× *g* for 2 min. BD Vacutainer^®^ SSTs were likewise allowed to clot for 30–60 min and separated by centrifugation at 1500× *g* for 10 min. Serum was removed from each Microtainer^®^ by pipette and weighed in a tube of known empty weight. Acceptance criteria for the Comfort Draw collection were 400 µL of blood and 100 µL of serum (following centrifugation).

A panel of 18 chemistry analytes plus hemolysis index were measured within 4 h of collection for each of the capillary and venous serum samples using Roche cobas^®^ 8000 autoanalyzers (Roche Diagnostics; Indianapolis, IN, USA): albumin, alkaline phosphatase (ALP), alanine aminotransferase (ALT), aspartate aminotransferase (AST), total bilirubin, blood urea nitrogen (BUN), calcium, carbon dioxide, cholesterol, chloride, creatinine, glucose, high-density lipoprotein (HDL), potassium, sodium, total protein, triglycerides, and C-Reactive Protein using a high-sensitivity assay (CRPhs). Low-density lipoprotein (LDL) levels were also determined (where possible) using an updated equation [15]. A list of the assays utilized can be found in Supplemental Table S1.

The absolute or relative biases of measurements from serum obtained with Comfort Draw were determined with respect to paired measurements from conventionally collected venous serum, and biases were compared to CLIA acceptance limits. For each analyte, percent agreement was determined by dividing the number of sample results within acceptance limits by the total number of results. The acceptable percent agreement for each analyte, as collected by Comfort Draw, was defined as greater than two-thirds (i.e., >66.7%) of individual sample results having an acceptable bias (within CLIA limits) with respect to the conventionally collected venous serum. As a secondary analysis, values for each analyte obtained with Comfort Draw versus conventional venous draw were plotted, and best-fit lines were generated by Deming regression using Prism 10.

## 3. Results

### 3.1. Vacuum Pressure and Skin Temperature

When compressed against the upper arm by a technician, Comfort Draw generated an average initial vacuum pressure of 20.7 kPa with a coefficient of variation (CV) of 6.8%. Vacuum pressure declined to 18.4 kPa with a CV of 12.4% after 4 min. Compared to the initial peak, average vacuum pressure declined by 7.0%, 8.6%, 9.6%, and 11.0% after 1 min, 2 min, 3 min, and 4 min of activation, respectively, indicating an acceptable loss of vacuum over the collection time (Supplemental Figure S2).

The optimal skin temperature to maximize capillary dilation for blood collection is about 42 °C, whereas the threshold for pain due to excessive temperature is about 43.5 °C [14]. Preliminary testing suggested that warm packs typically used for site prep for infant heel stick procedures achieved skin temperatures that were several degrees cooler than optimal. Therefore, a custom warmer was developed for use with Comfort Draw. The skin temperatures achieved by the custom warmer vs. the Medline Infant Heel Warmer after 1, 2, 3, and 4 min post-activation are shown in Supplemental Figure S3. When each warmer model was preconditioned at 22 °C, the custom prep warmer achieved a peak skin temperature of 41.1 °C versus 36.4 °C for the Medline infant heel warmer. When each warmer model was preconditioned at 25 °C, the custom warmer achieved a peak skin temperature of 42.2 °C versus 37.8 °C for the Medline infant heel warmer. For all models and conditions tested, temperature was stable within 2.0 °C throughout the duration of testing.

### 3.2. Blood Volume and Analyte Evaluation

**Figure 2:**
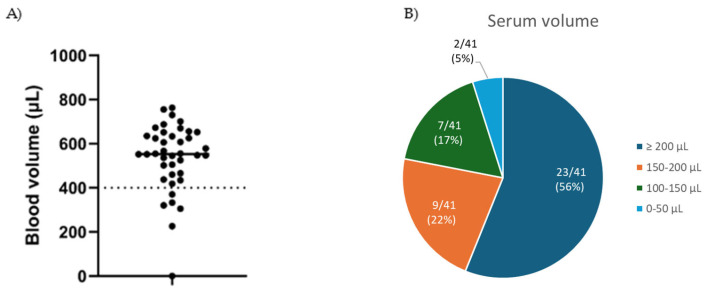
(**A**) Quantitative assessment of blood volume obtained from *n* = 41 Comfort Draw collections. The dotted line at 400 µL indicates the nominal minimum fill line for the BD Microtainer^®^ SST; (**B**) Categorical assessment of serum volume obtained from the blood specimens after centrifugation and extraction.

A measurable amount of blood was obtained from 41 out of 42 subjects, with 85.4% obtaining at least 400 µL (the Microtainer’s minimum fill volume) and 95.1% obtaining at least 100 µL of serum after centrifugation (Figure 2A,B). A mean volume of 538 µL of capillary blood was collected using the Comfort Draw method. A comparison of the blood volume collected using the Comfort Draw system versus fingerstick in the preliminary *n* = 12 study is provided as Supplemental Figure S4. The mean specimen hemolysis index was 4.1 for the capillary serum specimens, where volume was sufficient to test, vs. 4.9 for the venous serum. Summary statistics for blood specimen quality metrics are shown in Table 1.

Analytical measurements of the capillary blood specimens collected with Comfort Draw agreed (within CLIA acceptance limits) with corresponding measurements of venous specimens at least 66.7% of the time for all analytes tested other than glucose (56.8%) and potassium (61.5%) and had mean relative or absolute bias with respect to venous specimens within CLIA acceptance limits for all analytes except glucose. Table 2 shows CLIA acceptance limits for each analyte, mean absolute or relative bias for each analyte, and the proportion of individual specimens within CLIA limits. Supplemental Figure S5 shows individual specimen bias plots with respect to CLIA limits for each analyte. Supplemental Figure S6 shows regression plots with respect to CLIA limits for each analyte.

### 3.3. Pain Assessment

**Figure 3:**
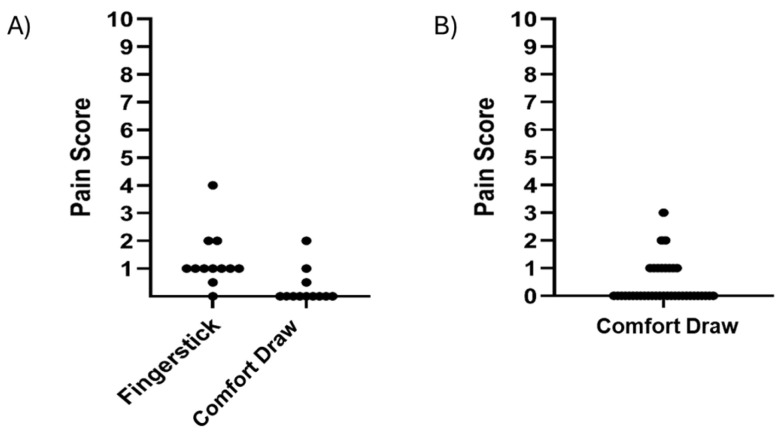
(**A**) Self-reported pain score on a scale of 0 (no pain) to 10 (worst imaginable) for the Comfort Draw system vs. fingerstick in the preliminary *n* = 12 subject comparison study. (**B**) Self-reported pain score from *n* = 38 clinical subjects on a scale of 0 (no pain) to 10 (worst imaginable).

In the preliminary comparison study with *n* = 12 subjects, pain from the Comfort Draw method (median value of 0) was rated significantly lower than pain from fingerstick (median value of 1) by the Mann–Whitney test (*p* = 0.0013; Figure 3A). In the larger clinical evaluation (*n* = 38), average subject-rated pain associated with the vacuum-assisted capillary draw procedure was 0.39 on a scale of 0 to 10, with 71.1% of subjects reporting a pain score of 0 (no pain) and 21.1% reporting a pain score of 1 (Figure 3B).

## 4. Discussion

In the preliminary clinical study, Labcorp Comfort Draw™ was found to be a valid method for collecting capillary blood specimens relative to conventionally collected venous samples for 17 out of the 19 analytes examined. A testable quantity of serum was obtained from 95.1% of subjects, and the majority of subjects self-reported no pain during the collection procedure. Specimen quality metrics, including collection success rate, average blood collection volume, serum yield, and hemolysis levels, were comparable to those previously reported for upper arm devices [5,6,10,11,12,16]. Likewise, the reported set of qualified analytes was comparable to previously reported comparisons between fingerstick and venous blood, as well as upper arm devices and venous blood [5]. As with previously studied methods of collecting capillary blood, glucose and potassium levels showed significant biases with respect to conventionally collected venous blood [6,17,18,19]. A direct comparison of Comfort Draw against alternative methods of capillary blood collection was outside the scope of this preliminary study. However, taken together, the data suggest that Comfort Draw is a viable assist device for collecting capillary blood for diagnostic purposes using conventional lancets.

This preliminary clinical study was conducted on healthy volunteers. Therefore, one limitation in the study design is that most analyte measurements were within healthy ranges. It is possible that interferences from the Comfort Draw device would be revealed at more extreme analyte values. The success rate should also be interpreted in the context of healthy adult subjects, whereas illness, advanced age, or pediatric use may decrease the success rate of capillary collection.

The Comfort Draw device is mechanically simple, comprising a single piece of molded silicone rubber. Each Comfort Draw device contains approximately 20 g of silicone, which has a material cost of less than USD 1 at high volumes [20]. The mass-produced conventional lancet used in this study is commercially available from a retail vendor for less than USD 2.50 each [21], compared to publicly listed retail prices of USD 30 for the Tasso+ device [22] and USD 19.99 for the TAP^®^ Micro Select device [23]. Bulk, direct-from-manufacturer purchase price for both the conventional lancets and the vacuum-assisted lancing devices is not publicly listed and would be significantly lower. However, the difference in lancet retail price suggests a potentially substantial cost advantage for the Comfort Draw system. In addition, unlike other upper arm devices with an integrated lancet function, Comfort Draw is not rendered inoperable after one use. Although the success rate for secondary draws has not yet been established, this feature provides the potential opportunity to collect additional blood tubes from a given subject using the same device, further improving the workflow and economics of capillary blood collection.

The simplicity of the Comfort Draw device does present some limitations. Compared to upper arm devices with integrated lancets, Comfort Draw requires an additional step of actuating the conventional lancet and aligning the Comfort Draw device with the resulting wound. Future studies should evaluate usability by untrained users (e.g., for home collection). Additionally, most upper arm devices include an adhesive ring to attach the device to the skin as opposed to the suction used to adhere the Comfort Draw device. This design simplification renders Comfort Draw more prone to vacuum leakage compared to other upper arm collection devices. On the other hand, Comfort Draw can be re-pumped as needed to restore the vacuum—an option not available with other devices when applied to difficult skin surfaces, such as areas with body hair.

Compared to fingerstick, Comfort Draw offers the advantages of reduced pain (being used on body surfaces with lower pain receptor density) and easier alignment of the collection tube with the blood flow path. Though Comfort Draw would remain a more costly option than traditional fingerstick, it may offer considerable cost savings compared to alternative upper arm collection devices.

Comfort Draw may help to address limitations in the currently available blood collection options. For example, Comfort Draw may provide a reduced-pain option for in-clinic collection of standard blood panels where the reimbursement rate does not support the use of more sophisticated devices. Comfort Draw could provide an alternative to venous phlebotomy for patients with difficult-to-access veins. It is estimated that 12% of patients exhibit difficult venous access (DVA), often requiring two or more sticks during venipuncture attempts [24]. Populations with a high prevalence of DVA include the elderly, the chronically ill, and pediatric populations [25]. Difficulties experienced during venipuncture or venous access can result in pain and patient dissatisfaction with the health care facility [26]. As a relatively economical blood collection method that bypasses the need for venipuncture, Comfort Draw may, therefore, help improve access to diagnostic testing and meaningfully improve patient experience for sensitive populations.

## 5. Patents

Labcorp has filed an International Patent Application (PCT/US2024/058491) and a U.S. Patent Application (No. 18/968,942) covering the Comfort Draw device discussed in this manuscript; G.J.S. and U.Y.S. are listed inventors on this patent.

## Figures and Tables

**Table 1:** Quality metrics for blood specimens collected by Comfort Draw.

Specimen Quality Metric	Acceptance Criteria	Proportion of Samples Meeting Criteria
Blood volume	>400 µL	85.4% (35/41)
Serum volume	>100 µL	95.1% (39/41)
Hemolysis index	<40	100.0% (39/39)

**Table 2:** CLIA acceptance limits, mean absolute or relative bias, and proportion of individual specimens within CLIA limits for each analyte. Values in green were within limits; values in red were not within limits.

Analyte	CLIA Limit	Mean Result (Control)	Mean Result (Comfort Draw)	Mean Bias	Percentage of Specimens Within CLIA Limits
Albumin	±8.0%	4.47 g/dL	4.54 g/dL	+1.6%	97.4%
ALP	±20.0%	59.8 U/L	60.2 U/L	+1.2%	100%
ALT	±15.0%	22.6 U/L	22.6 U/L	−0.1%	83.8%
AST	±15.0%	20.1 U/L	20.3 U/L	+1.8%	84.2%
Total Bilirubin	±20.0%	0.40 mg/dL	0.37 mg/dL	−7.0%	92.3%
BUN	±9.0%	12.7 mg/dL	13.2 mg/dL	+3.4%	74.4%
Calcium	±1.0 mg/dL	9.57 mg/dL	9.49 mg/dL	−0.08 mg/dL	100%
Carbon Dioxide	±20.0%	24.4 mmol/L	20.10 mmol/L	−17.7%	75%
Chloride	±5.0%	102.1 mmol/L	104.8 mmol/L	+2.6%	100%
Cholesterol	±10.0%	179.0 mg/dL	180.9 mg/dL	+1.0%	100%
Creatinine	±10.0%	0.912 mg/dL	0.834 mg/dL	−5.3%	84.2%
Glucose	±8.0%	95.4 mg/dL	103.6 mg/dL	+8.6%	56.8%
HDL	±20.0%	52.5 mg/dL	52.8 mg/dL	+0.6%	100%
LDL (calculated)	±20.0%	101.1 mg/dL	101.4 mg/dL	+0.3%	100%
Potassium	±0.3 mmol/L	4.32 mmol/L	4.57 mmol/L	+0.26 mmol/L	61.5%
Sodium	±4.0 mmol/L	138.0 mmol/L	137.8 mmol/L	−0.22 mmol/L	100%
Total Protein	±8.0%	7.20 g/dL	7.27 g/dL	+1.0%	100%
Triglycerides	±15.0%	123.1	129.0	+4.8%	91.7%
CRPhs	±30.0%	5.11 mg/L	5.22 mg/L	+2.3%	100%

## Data Availability

The Dataset is available on request from the authors.

## Supplementary Materials

The following supporting information can be downloaded at: https://www.mdpi.com/article/10.3390/diagnostics15151935/s1.

## Abbreviations

The following abbreviations are used in this manuscript:

ALP	Alkaline phosphatase
ALT	Alanine aminotransferase
AST	Aspartate aminotransferase
BUN	Blood urea nitrogen
CRPhs	C-Reactive Protein using a high-sensitivity assay
CV	Coefficient of variation
HDL	High-density lipoprotein
LDL	Low-density lipoprotein
