# Supplementary material for: Evaluation of a Simplified Upper Arm Device for Vacuum-Assisted Collection of Capillary Blood Specimens
Source: Diagnostics (Basel). 2025 Jul 31;15(15):1935. doi: 10.3390/diagnostics15151935 (PMC12345872; doi:10.3390/diagnostics15151935)
Supplement: Supplementary file 1 [file diagnostics-15-01935-s001.zip › diagnostics-3737710-supplementary.pdf]

Supplemental Table S1: Details of the Roche assays utilized, along with maximum allowable hemolysis indices for serum-based analytes.

Analyte	CLIA Acceptance Limits **	Methodology*	Maximum Allowable Hemolysis Index (mg/dL)
Albumin	±8%	Colorimetric	1000
ALP	±20%	Colorimetric	200
ALT	±15% or ±6 IU/L	Colorimetric	90
AST	±15% or ±6 IU/L	Colorimetric	40
Bilirubin, total	±20% or ±0.4 mg/dL	Colorimetric	1000
BUN	±9% or ±2 mg/dL	Colorimetric	1000
Calcium	±1.0 mg/dL	Colorimetric	1000
Carbon Dioxide	±20%	Colorimetric	600
Chloride	±5%	Ion selective electrode	1000
Cholesterol	±10%	Colorimetric	700
Creatinine (Jaffe)	±10% or ±0.2 mg/dL	Colorimetric	1000
Glucose	±8% or ±6 mg/dL	Colorimetric	1000
HDL	±20% or ±6 mg/dL	Colorimetric	1200
Potassium	±0.3 mmol/L	Ion selective electrode	"Avoid"
Protein, total	±8%	Colorimetric	500
Sodium	±4 mmol/L	Ion selective electrode	1000
Triglycerides	±15%	Colorimetric	700
CRPhs	±30% or ±1 mg/L	Immunoturbidimetric	1000

**Additional details can be found in the respective package inserts listed below.*

***Acceptance limits were defined by the minimum allowable bias based on biological variability.*

List of Roche references and materials utilized

1. Roche Diagnostics, Indianapolis, IN, cobas® 8000 modular analyzer series, Operator's Manual Version 5.6, Software Version 06-08, copyright 2021.
2. Roche Diagnostics, Indianapolis, IN, ALB2 – Albumin Gen. 2 Method Sheet (05166861190), Version: 2024-06, V8.0 English.
3. Roche Diagnostics, Indianapolis, IN, ALP2 – Alkaline Phosphatase acc. to IFCC Gen. 2 Method Sheet (05166888190), Version: 2021-10, V9.0 English.

4. Roche Diagnostics, Indianapolis, IN, ALT – Alkaline aminotransferase acc. to IFCC without pyridoxal phosphate activation Method Sheet (05850797190), Version: 2023-09, V10.0 English.
5. Roche Diagnostics, Indianapolis, IN, AST – Aspartate aminotransferase acc. to IFCC without pyridoxal phosphate activation Method Sheet (05850819190), Version: 2022-10, V9.0 English.
6. Roche Diagnostics, Indianapolis, IN, UREAL – Urea/BUN Method Sheet (05171873190), Version: 2024-07, V7.0 English.
7. Roche Diagnostics, Indianapolis, IN, CA2 – Calcium Gen. 2 Method Sheet (05168449190), Version: 2023-09, V9.0 English.
8. Roche Diagnostics, Indianapolis, IN, CO2-L – Bicarbonate Liquid Method Sheet (05446376191), Version: 2022-09, V14.0 English.
9. Roche Diagnostics, Indianapolis, IN, ISE indirect Na, K, Cl for Gen. 2 Method Sheet, Version: 2024-05, V11.0 English.
10. Roche Diagnostics, Indianapolis, IN, CHOL2 – Cholesterol Gen. 2 Method Sheet (05168538190), Version: 2023-10, V13.0 English.
11. Roche Diagnostics, Indianapolis, IN, CREJ2 – Creatinine Jaffé Gen. 2 Method Sheet (06407137190), Version: 2023-11, V17.0 English.
12. Roche Diagnostics, Indianapolis, IN, GLUC3 – Glucose HK Gen. 3 Method Sheet (05168791190), Version: 2024-07, V11.0 English.
13. Roche Diagnostics, Indianapolis, IN, HDLC4 – HDL-Cholesterol Gen. 4 Method Sheet (072528582190), Version: 2024-08, V3.0 English.
14. Roche Diagnostics, Indianapolis, IN, BILT3 – Bilirubin Total Gen. 3 Method Sheet (05795419190), Version: 2024-01, V10.0 English.
15. Roche Diagnostics, Indianapolis, IN, SI2 – Serum Index Gen. 2 Method Sheet (05172179190), Version: 2024-03, V7.0 English.
16. Roche Diagnostics, Indianapolis, IN, TP2 – Total Protein Gen. 2 Method Sheet (05171385190), Version: 2023-09, V14.0 English.
17. Roche Diagnostics, Indianapolis, IN, TRIGL – Triglycerides Method Sheet (05171407190), Version: 2024-04, V13.0 English.
18. Roche Diagnostics, Indianapolis, IN, CRPHS – Cardiac C-Reactive Protein (Latex) High Sensitive Method Sheet (04628918190), Version: 2024-04, V15.0 English.

Supplemental Figure S1:

1. **Prepare materials**: unpack supplies, remove components from packaging, remove pin from lancet, and activate warm pack.

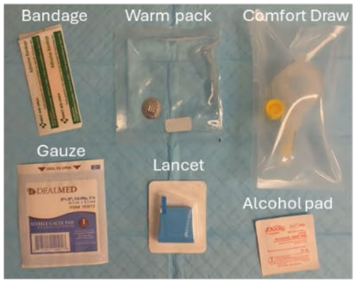

2. Warm the site: hold the activated warm pack against the upper arm collection site for 3 minutes.

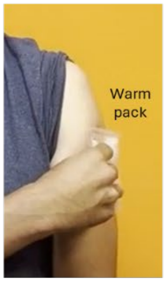

3. Clean the site and activate lancet: wipe collection site with an alcohol pad, hold the lancet against the skin, and press the button on the lancet to activate the device.

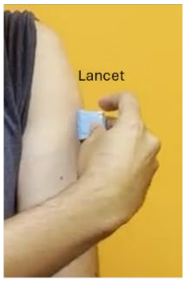

4. Activate Comfort Draw: center the Comfort Draw over the drop of blood, firmly compress the bulb and let go. If bulb reinflates during collection, compress the bulb again to maintain suction. Leave the device on the arm for approximately 4 minutes, or until the blood volume reaches the maximum fill line (600 μL).

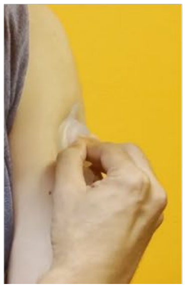

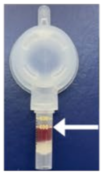

5. Remove Comfort Draw: pull the Comfort Draw off the arm using the tab; remove the tube from the bulb and cap the tube. Clean arm with gauze and apply bandage to collection site.

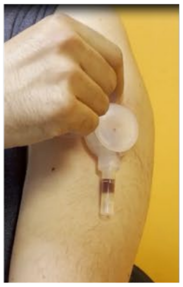

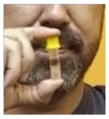

Supplemental Figure S2: Vacuum pressure (ambient pressure minus internal pressure) of Comfort Draw™ measured for 6 subjects in duplicate initially and once per minute over the course of 4 minutes

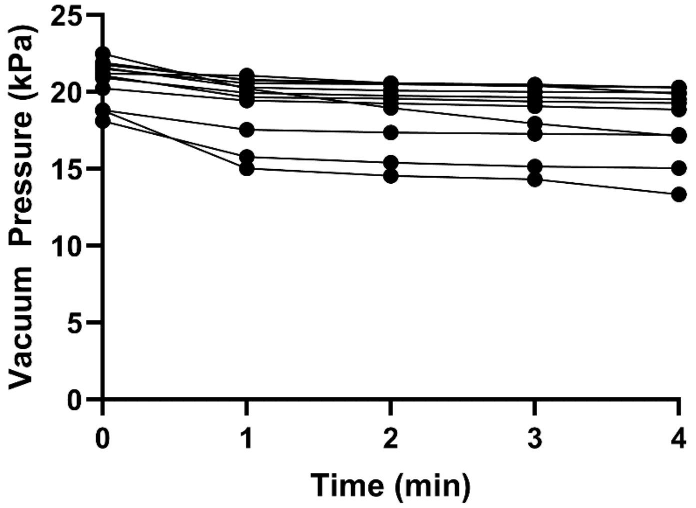

Supplemental Figure S3: Skin surface temperature measured at 1, 2, 3, and 4 minutes for a Medline Infant Heel Warmer versus the Comfort Draw prep warmer when preconditioned at 22 ° C or 25 ° C.

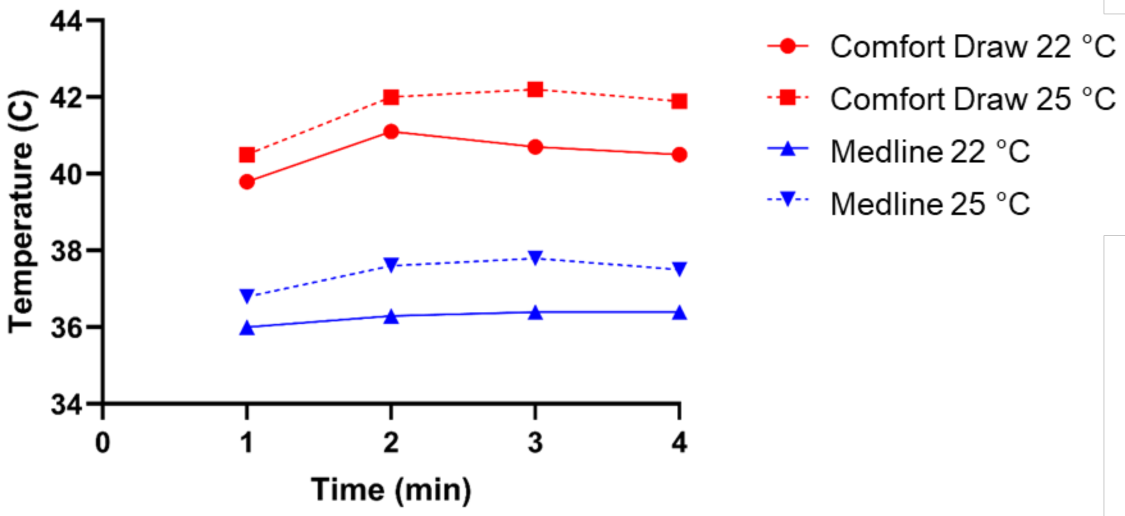

Supplemental Figure S4: Blood volume collected using the Comfort Draw system versus fingerstick in the initial n=12 comparison study.

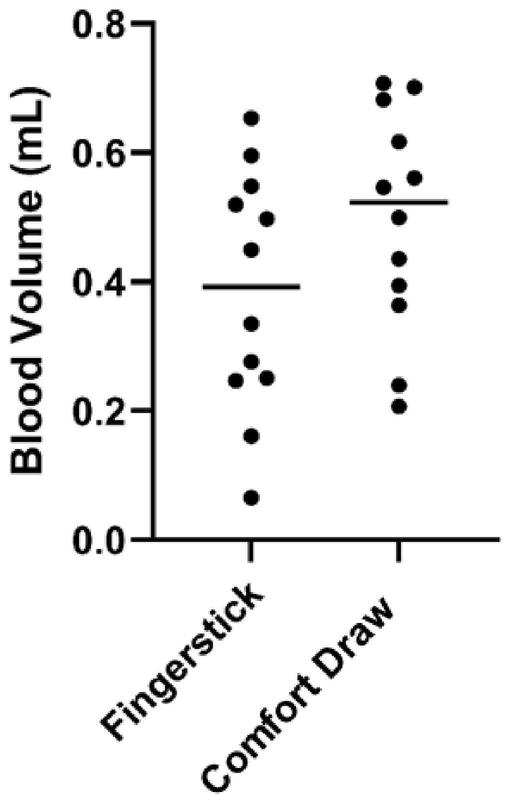

Supplemental Figure S5: Bias plots with respect to CLIA limits for each analyte. (Dotted lines represent CLIA limits for individual analytes.)

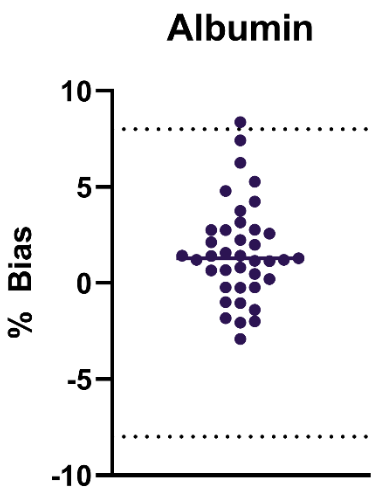

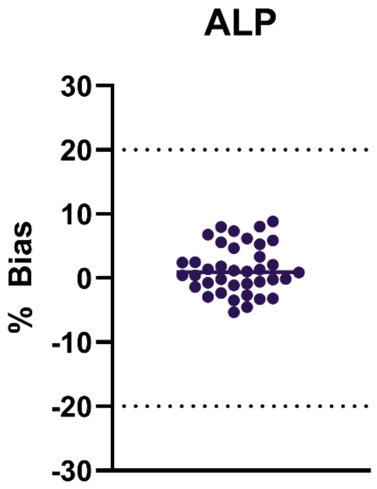

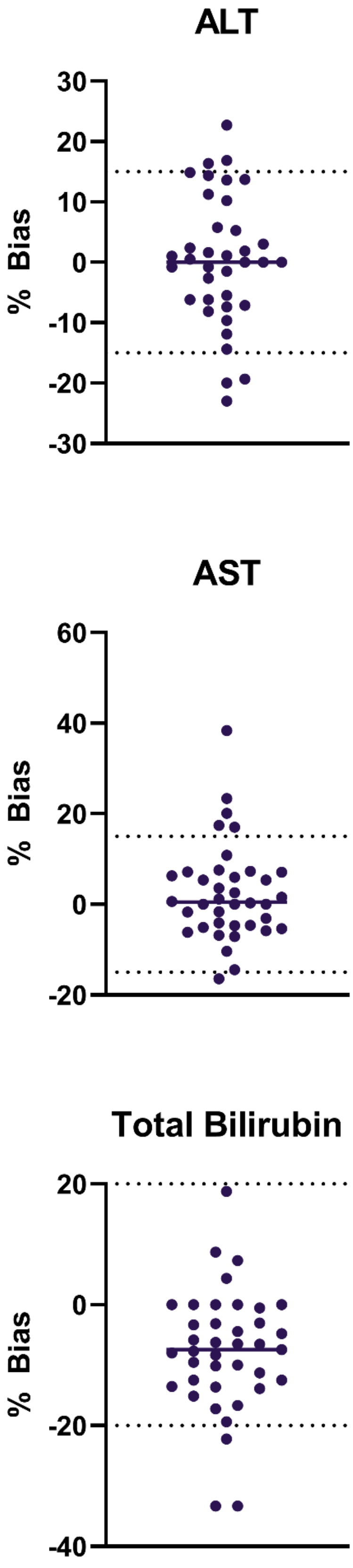
ALT
% Bias
30
20
10
0
-10
-20
-30
AST
% Bias
60
40
20
0
-20
Total Bilirubin
% Bias
20
0
-20
-40

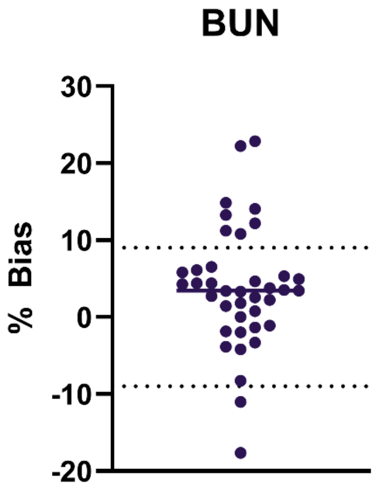
BUN
% Bias
30
20
10
0
-10
-20

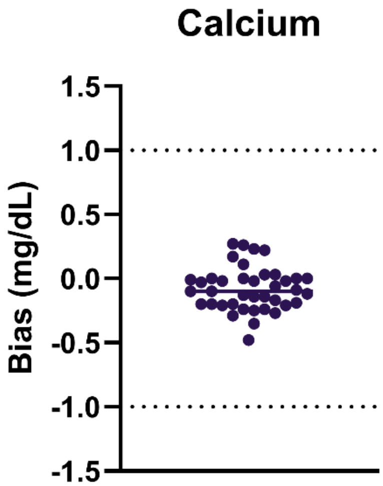
Calcium
Bias (mg/dL)
1.5
1.0
0.5
0.0
-0.5
-1.0
-1.5

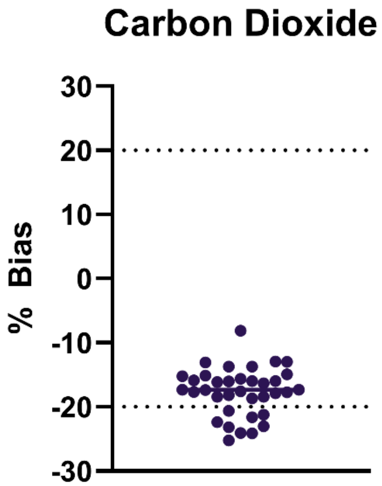
Carbon Dioxide
% Bias
30
20
10
0
-10
-20
-30

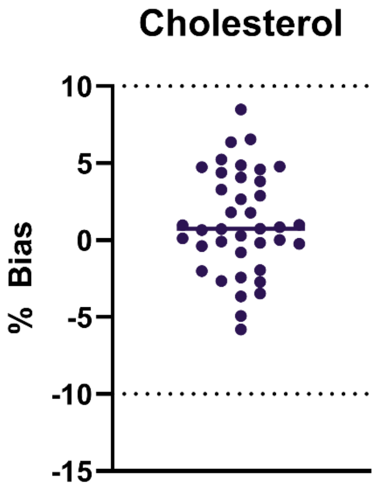
Cholesterol
% Bias
10
5
0
-5
-10
-15

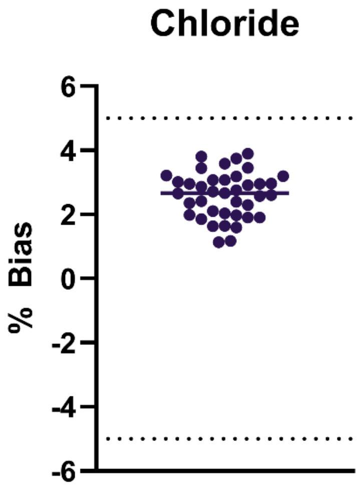
Chloride
% Bias
6
4
2
0
-2
-4
-6

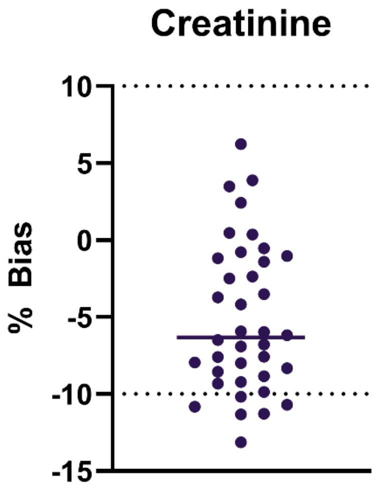
Creatinine
% Bias
10
5
0
-5
-10
-15

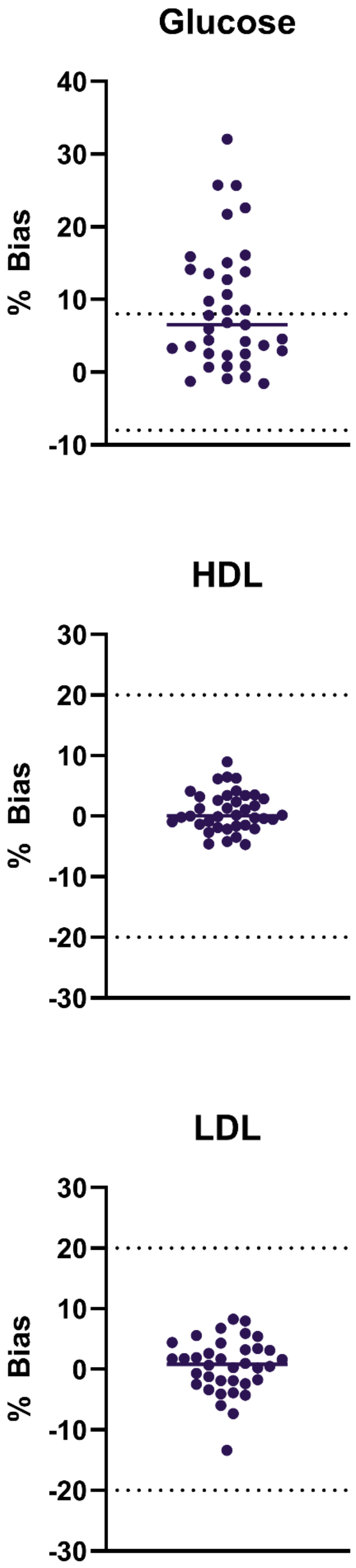
Glucose
40
30
20
10
0
-10
% Bias
HDL
30
20
10
0
-10
-20
-30
% Bias
LDL
30
20
10
0
-10
-20
-30
% Bias

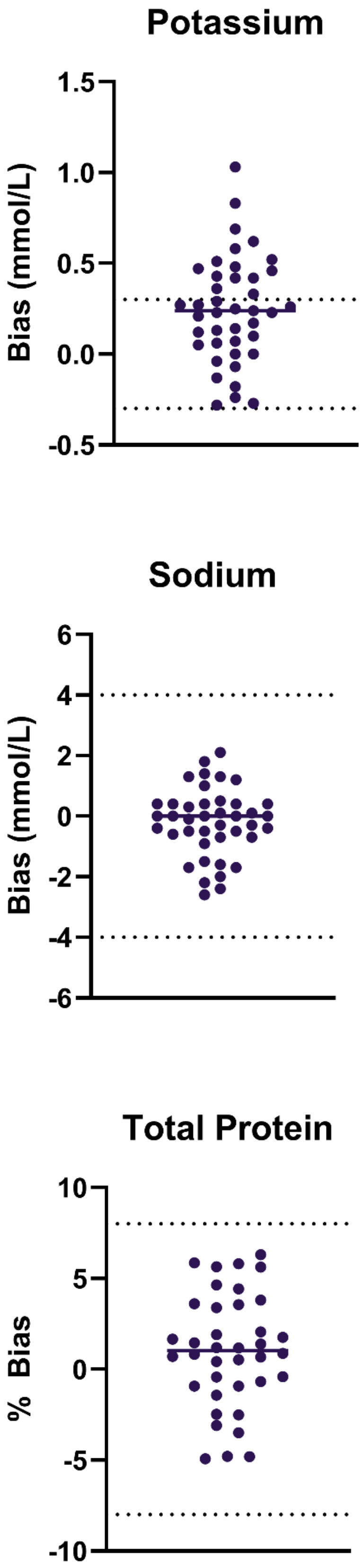
Potassium
Bias (mmol/L)
1.5
1.0
0.5
0.0
-0.5
Sodium
Bias (mmol/L)
6
4
2
0
-2
-4
-6
Total Protein
% Bias
10
5
0
-5
-10

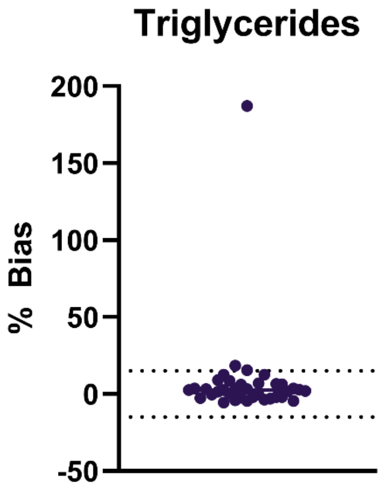
Triglycerides
200
150
100
50
0
-50
% Bias

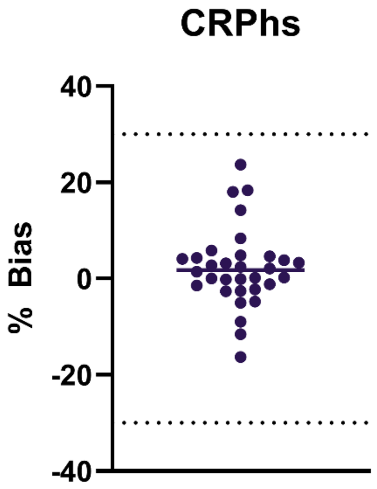
CRPhs
40
20
0
-20
-40
% Bias

Supplemental Figure S6: Regression plots with respect to CLIA limits for each analyte. (Dotted lines represent confidence intervals.)

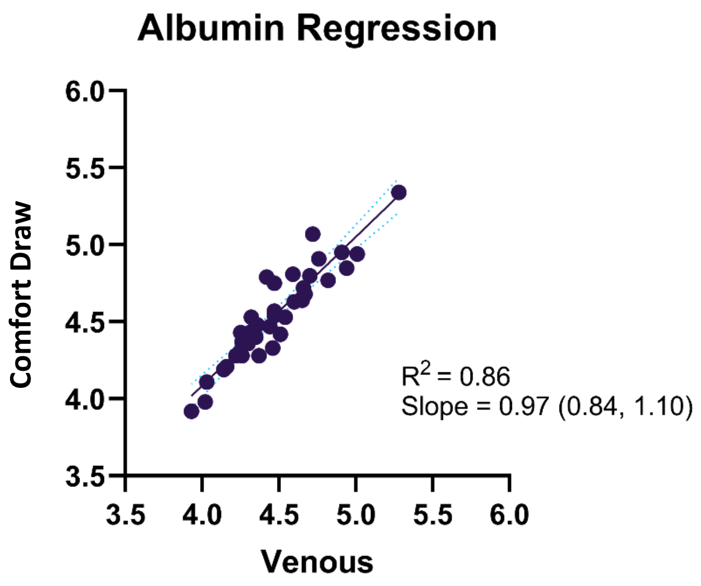

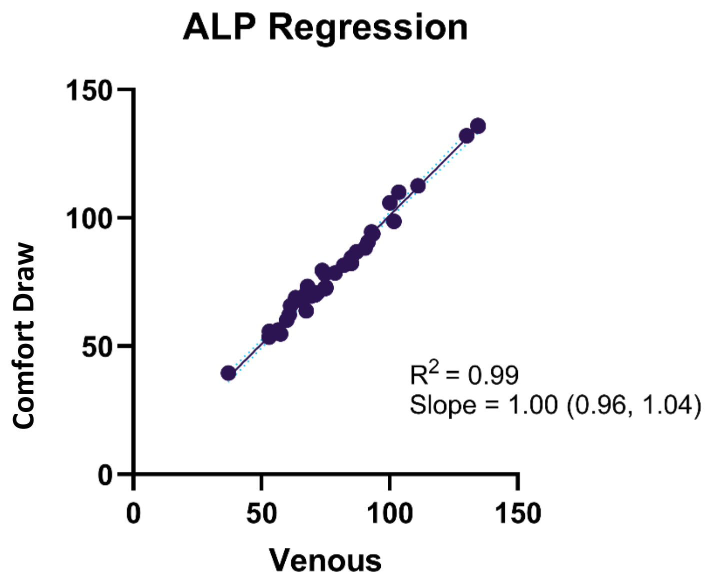

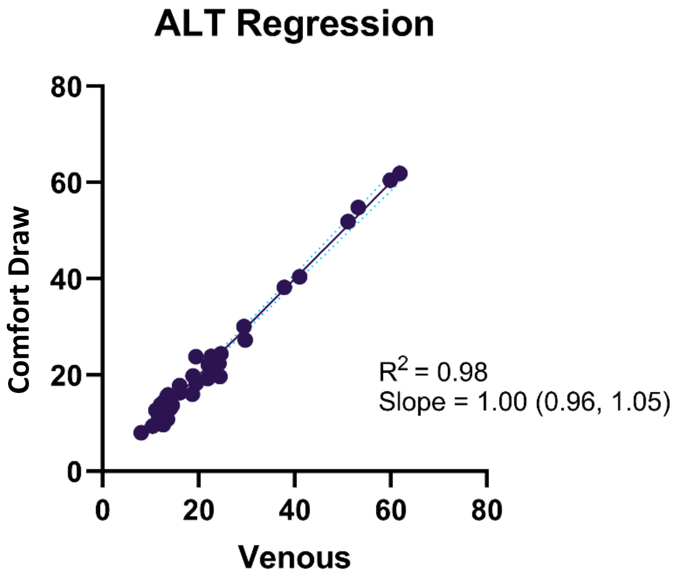
ALT Regression
Comfort Draw
Venous
0
20
40
60
80
R2 = 0.98
Slope = 1.00 (0.96, 1.05)

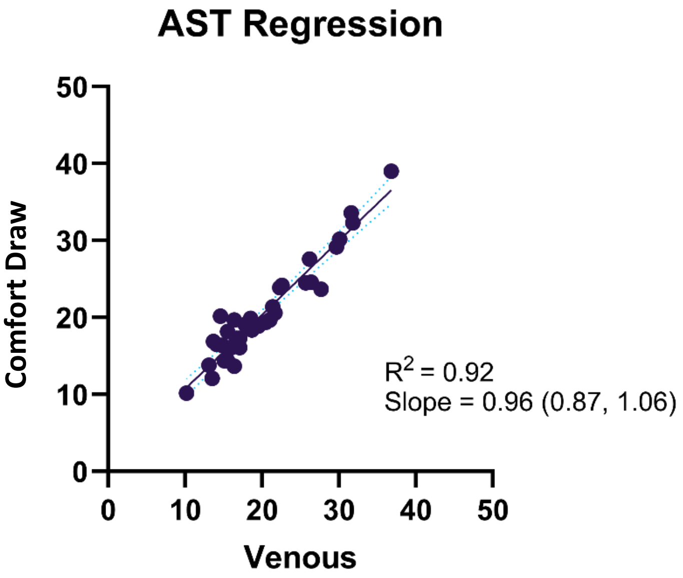
AST Regression
Comfort Draw
Venous
0
10
20
30
40
50
R2 = 0.92
Slope = 0.96 (0.87, 1.06)

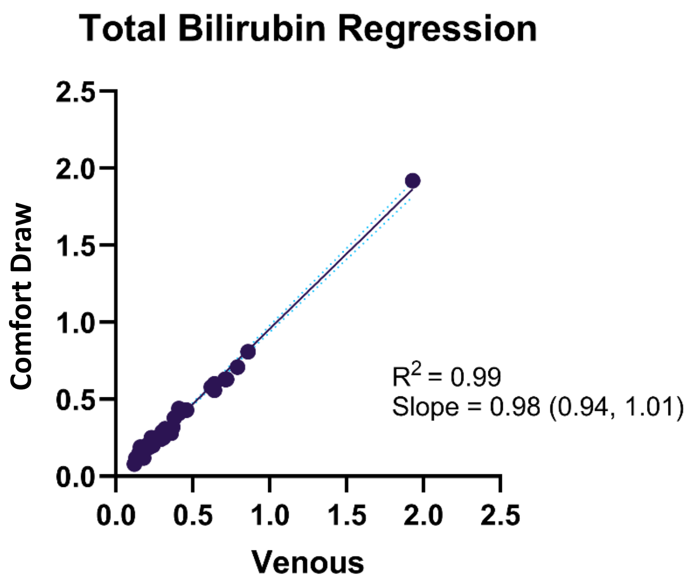
Total Bilirubin Regression
Comfort Draw
Venous
0.0
0.5
1.0
1.5
2.0
2.5
R^2 = 0.99
Slope = 0.98 (0.94, 1.01)

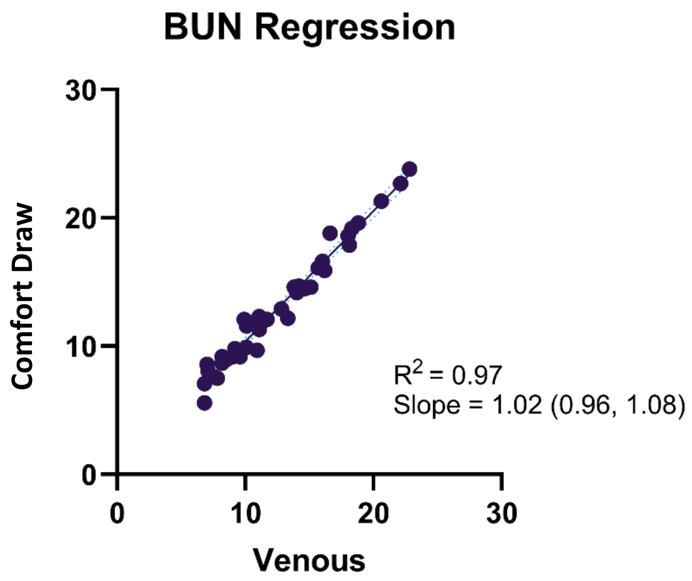
BUN Regression
Comfort Draw
Venous
0
10
20
30
R^2 = 0.97
Slope = 1.02 (0.96, 1.08)

Calcium Regression

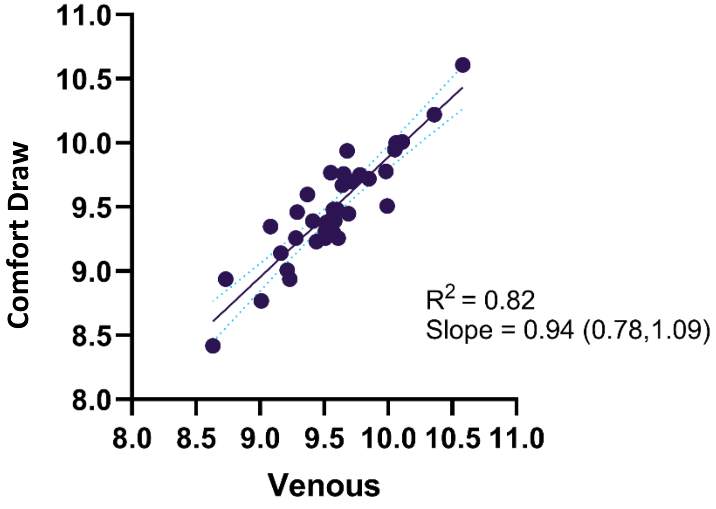

Carbon Dioxide Regression

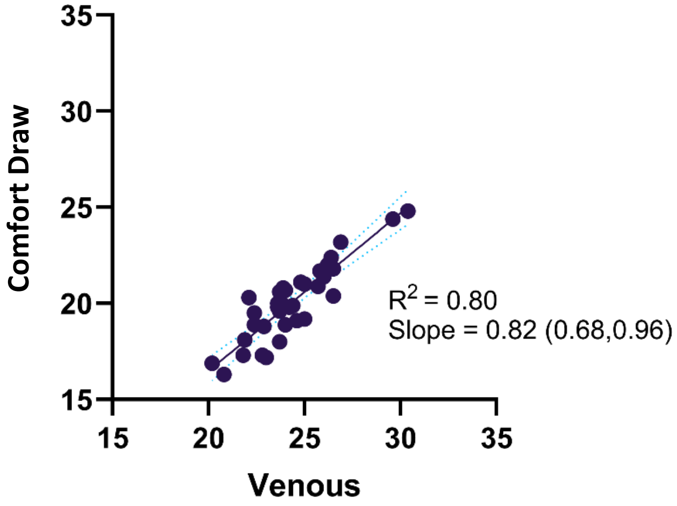

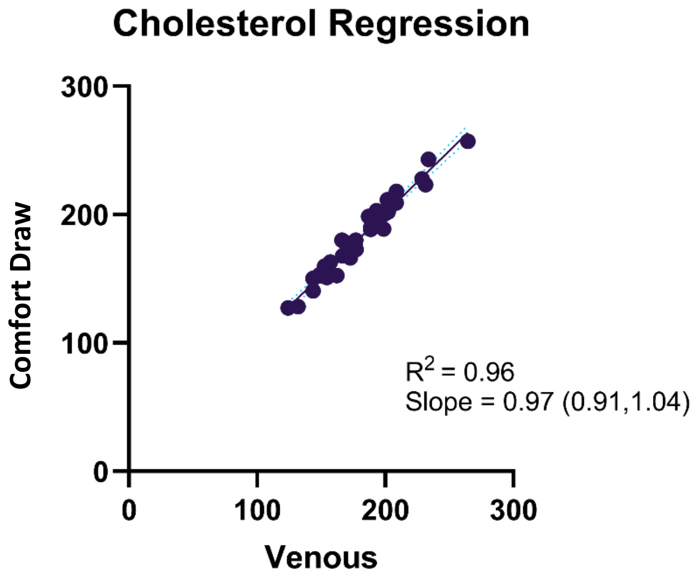
Cholesterol Regression
300
200
100
0
0
100
200
300
Comfort Draw
Venous
R2 = 0.96
Slope = 0.97 (0.91,1.04)

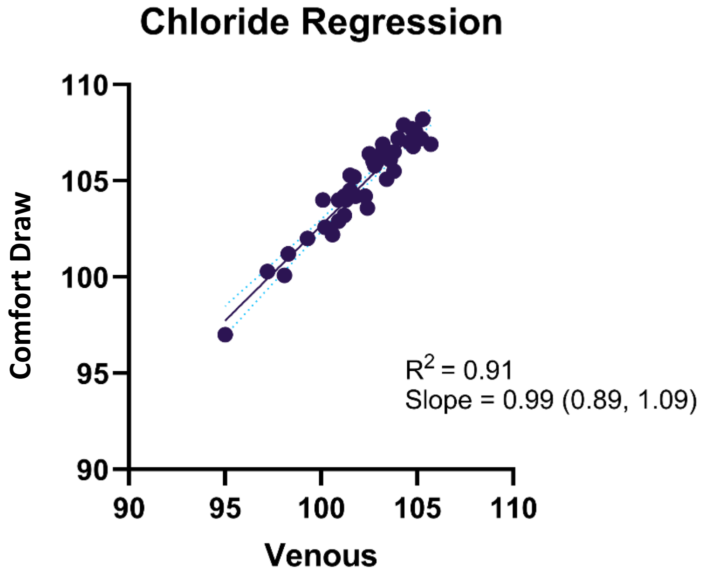
Chloride Regression
110
105
100
95
90
90
95
100
105
110
Comfort Draw
Venous
R2 = 0.91
Slope = 0.99 (0.89, 1.09)

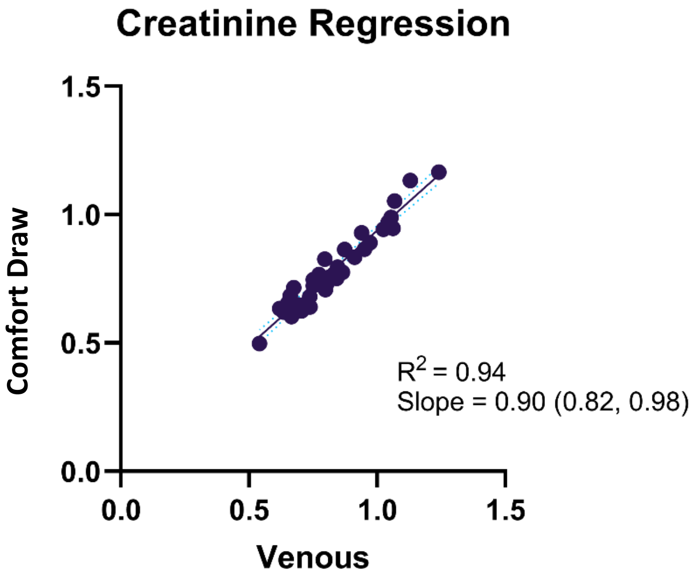
Creatinine Regression
Comfort Draw
Venous
1.5
1.0
0.5
0.0
0.0
0.5
1.0
1.5
R^2 = 0.94
Slope = 0.90 (0.82, 0.98)

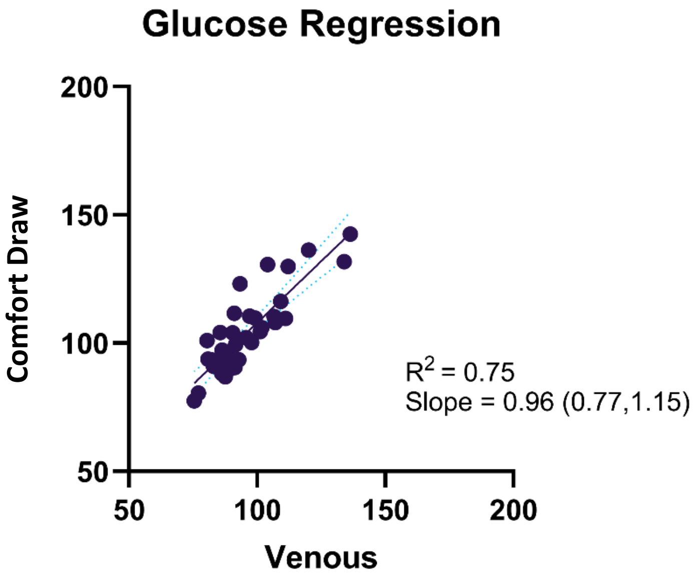
Glucose Regression
Comfort Draw
Venous
200
150
100
50
50
100
150
200
R^2 = 0.75
Slope = 0.96 (0.77,1.15)

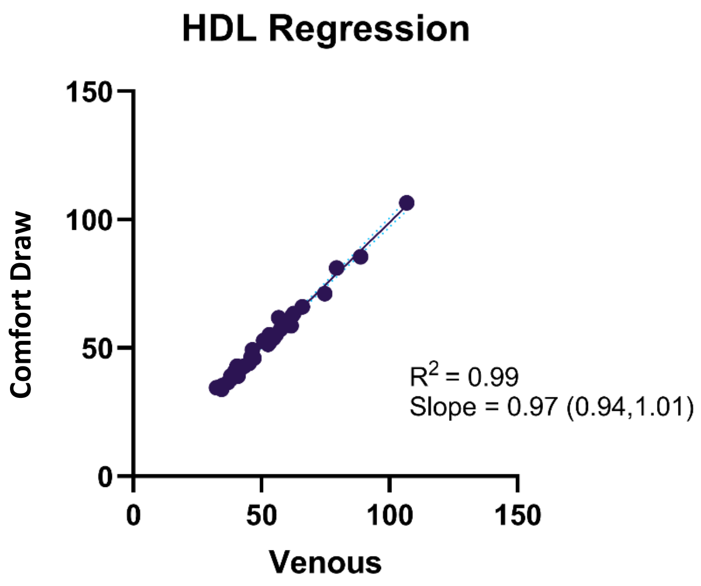
HDL Regression
150
100
50
0
0
50
100
150
Comfort Draw
Venous
R^2 = 0.99
Slope = 0.97 (0.94,1.01)

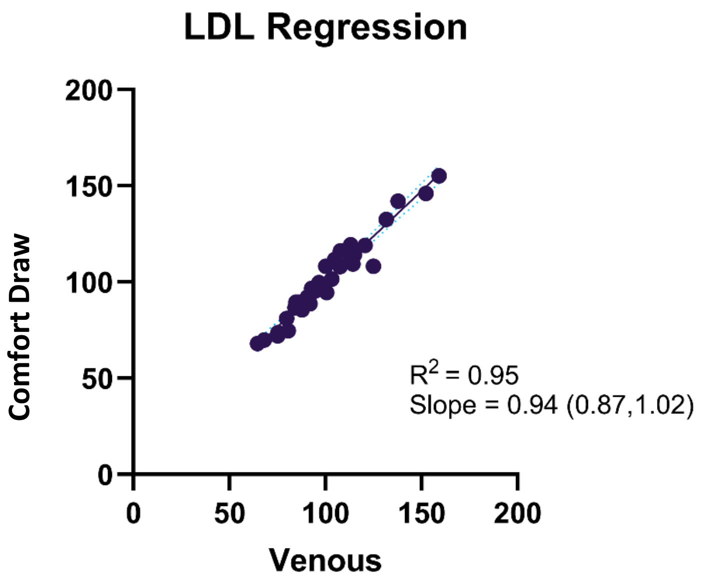
LDL Regression
200
150
100
50
0
0
50
100
150
200
Comfort Draw
Venous
R^2 = 0.95
Slope = 0.94 (0.87,1.02)

Potassium Regression

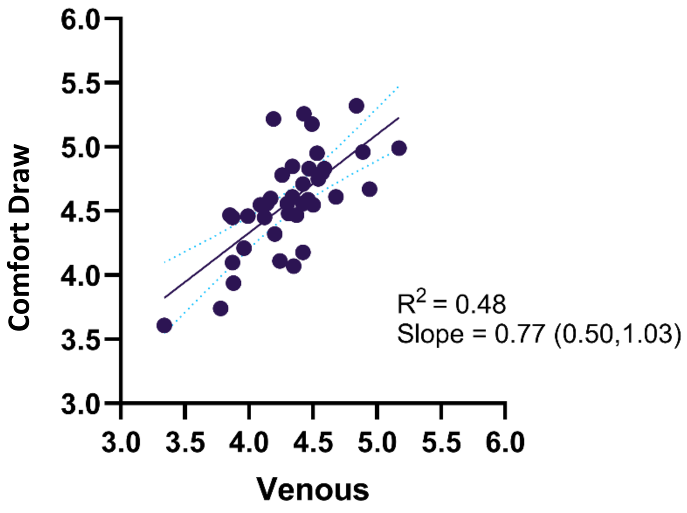

Sodium Regression

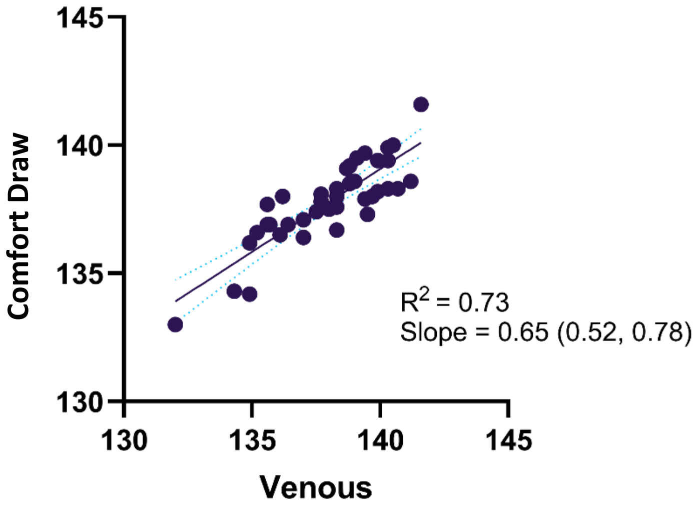

Total Protein Regression

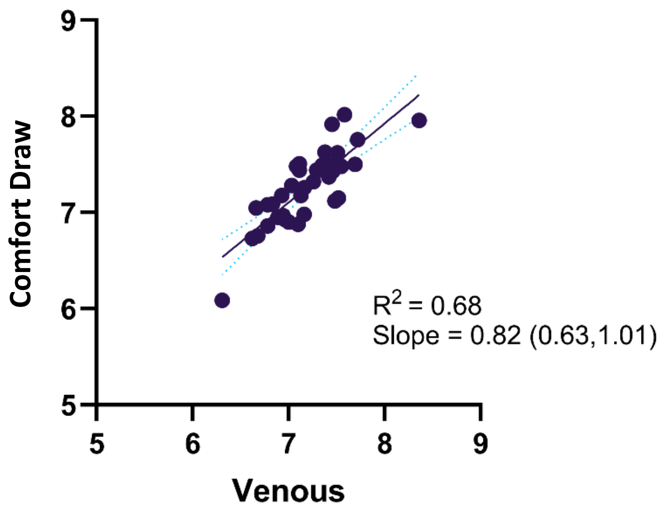

Triglycerides Regression

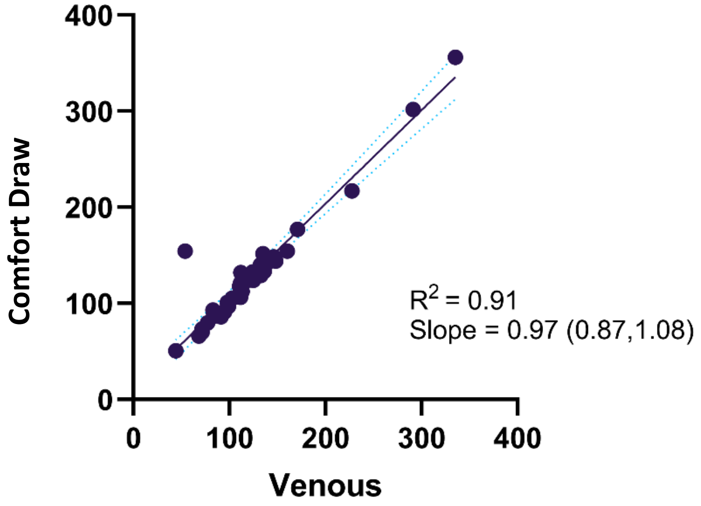

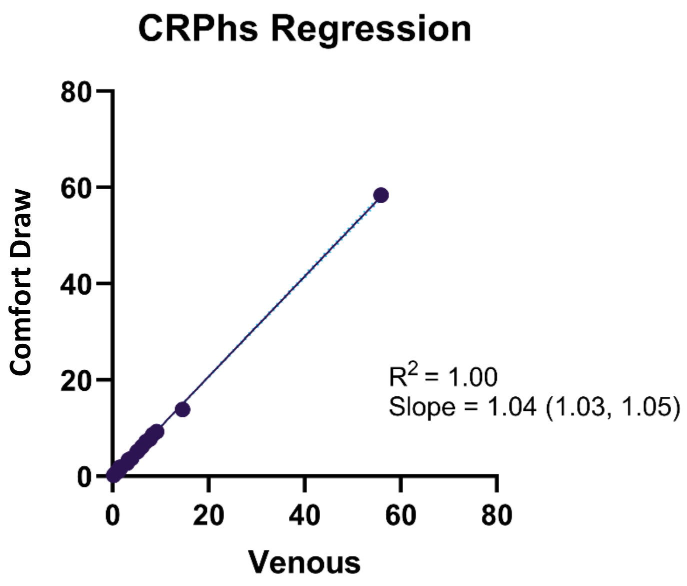
CRPhs Regression
Comfort Draw
Venous
0
20
40
60
80
0
20
40
60
80
R^2 = 1.00
Slope = 1.04 (1.03, 1.05)
